# A Large-Scale Analysis of the Relationship of Synonymous SNPs Changing MicroRNA Regulation with Functionality and Disease

**DOI:** 10.3390/ijms161023545

**Published:** 2015-09-30

**Authors:** Yuchen Wang, Chengxiang Qiu, Qinghua Cui

**Affiliations:** 1 Department of Biomedical Informatics, School of Basic Medical Sciences, Peking University, Beijing 100191, China; E-Mails: wangyuchen@bjmu.edu.cn (Y.W.); qiuchengxiang@bjmu.edu.cn (C.Q.); 2 Center for Noncoding RNA Medicine, Peking University Health Science Center, Beijing 100191, China; 3 MOE Key Lab of Molecular Cardiovascular Science, Peking University, Beijing 100191, China

**Keywords:** microRNA, synonymous SNP, functionality

## Abstract

Historically, owing to not changing amino acid composition of protein sequences, synonymous mutations are commonly assumed to be neutral during evolution and therefore have no effect on the phenotype and disease. Here, based on observations from large-scale analysis of genomic data, we predicted the putative synonymous SNPs that could result in functional consequences and disease risk through changing the microRNA-mediated gene regulation. We found that nearly half of the synonymous SNPs could affect protein expression by changing microRNA regulation in human genome and these SNPs significantly prefer to be associated with human diseases and traits. The synonymous SNPs changing microRNA-mediated gene regulation tend to be more under recent positive selection, prefer to affect gene expression, and implicate in human disease. We conclude that the miRNA-mediated regulation changes could be a potential mechanism for the contributions of synonymous SNPs to protein functions and disease risks.

## 1. Introduction

Genetic mutations play critical roles in various normal and abnormal biological processes, including evolution, phenotype, and disease. Traditionally, proteins are considered to be the centric functional molecules in molecular biomedicine [1] and therefore historically most of the efforts have focused on the mutations that can change the amino acid composition of protein sequences (non-synonymous mutations) [2], which are considered to have the potential to affect protein functions. Owing to not changing the protein sequences, synonymous mutations are commonly assumed to be neutral during evolution [3] and therefore would have no effect on the fitness of an organism and would have no roles in determining phenotype and disease [2]. However, recent efforts revealed that there is a codon usage bias for some synonymous mutation sites, suggesting that synonymous mutations could be not neutral and have functional consequences [3]. Moreover, recent advances have revealed several mechanisms to understand how synonymous mutations result in functional consequences. For example, synonymous mutations could change mRNA splicing [4], mRNA stability [5], protein translation efficiency, and protein folding [2]. These findings provided direct or indirect evidence for the functionality of synonymous mutations. However, these findings mainly resulted from specific case studies and can only interpret a limited number of synonymous mutations. For example, only the synonymous mutations near the exon-intron junction sites could change mRNA splicing. As a result, the mechanism of most of the functional synonymous SNPs revealed by recent large-scale GWAS (genome-wide association study) experiments [6] remains largely unclear. Here, we are wondering whether there are more general mechanisms to interpret the functional consequences and disease risk of synonymous mutations. For this purpose, based on previous wet-lab observations and our bioinformatics analysis, here we proposed that a large number of synonymous SNPs could result in functional consequences and disease risk through changing protein expression level by aberrant microRNA (miRNA)-mediated gene regulation.

MiRNAs are one class of small noncoding RNA molecules, which are ~22 nucleotides in length and mainly repress the gene expression at the post-transcriptional level by guiding the the RNA-induced silencing complex (RISC) to bind with target RNAs [7]. Up to now, nearly 2000 miRNAs (miRBase Release 19) have been identified in the human genome [8] and one miRNA could regulate hundreds of target genes. Increasing evidence has shown that miRNAs play critical roles in a variety of important biological processes. In theory, miRNAs could bind with various RNA molecules and therefore could be a bridge connecting the RNA world through competing interaction between RNAs and miRNAs [9]. For their importance, it is not surprising that miRNAs have been implicated in various human diseases [10], including cancer [11] and cardiovascular disease [12]. According to HMDD (Human microRNA Disease Database), currently ~400 human diseases have been reported to be associated with miRNAs [10], suggesting that miRNAs have a strong connection to human disease.

For miRNA-target interactions, some canonical rules have been established through numerous experiments during the past decade [13]. One critical canonical rule is that miRNAs interact with their targets mainly through the base-pair binding of their seed regions with the 3ʹUTR of target mRNAs [14]. However, there are a few cases for exceptions to the above rule. The miRNA-binding sites at mRNAs have occasionally been reported in 5ʹ UTR [15] and coding regions [16]. A recent study revealed that a synonymous SNP in IRGM alters a binding site for miR-196 and caused deregulation of IRGM-dependent xenophagy in Crohn’s disease [16]. This finding suggests that synonymous SNPs mediated changes of miRNA regulation could be a potential mechanism of functional consequences and disease risk. Strikingly, two recent studies identified a large number (40%–60%) of miRNA-binding sites located in the coding regions of mRNAs by high-throughput experiments [13,17], suggesting that for the SNPs located in the coding regions (including synonymous SNPs and non-synonymous SNPs) mediating changing of miRNA regulation is not occasional but frequent. This implies a putatively general model for the contributions of synonymous SNPs to functional consequences and disease risks. Here, based on large-scale bioinformatic analysis, we supported indirect quantitative evidence for this model.

## 2. Results

### 2.1. The Putative Model for the Contributions of Synonymous Mutations to Functionality and Disease by miRNA-Mediated Gene Regulation

**Figure 1:**
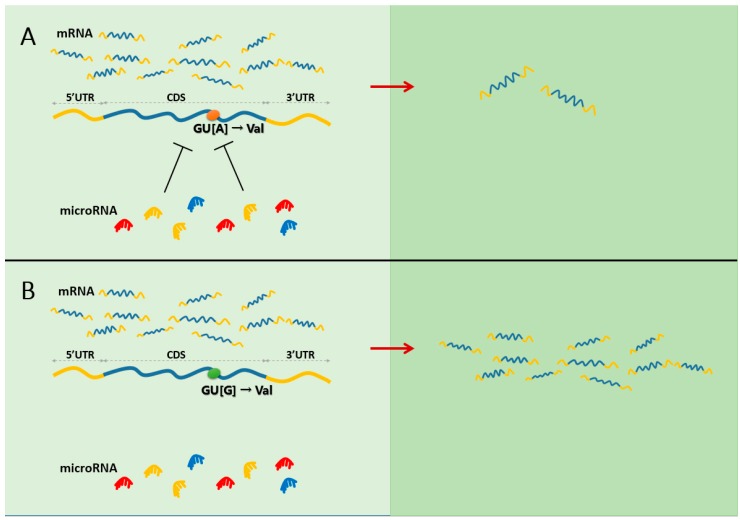
The model for the mechanism that synonymous SNPs could have functional consequences through aberrant miRNA-mediated gene regulation. (**A**) An “A” mutant (the orange nucleotide) at the synonymous SNPs in one gene makes it possible that the gene is regulated by the given miRNA, which will result in decreased gene product; (**B**) As a comparison, the gene with a “G” mutant (the green nucleotide) cannot be regulated by the given miRNA, which have no effect on gene product.

Based on the above observation, Figure 1 shows the putative model that generally synonymous SNPs could contribute to functional consequences and disease risks through changing the miRNA binding with the coding regions of target genes.

As shown in Figure 1, although the synonymous SNP does not result in changes of protein amino acid (both GUA and GUG code Val), the miRNA-regulation to the mRNAs the two alleles (A/G) are different. For mRNAs with allele A, they can be regulated by the given miRNAs (Figure 1A); whereas the mRNAs with allele G deleted the regulation by the given miRNAs (Figure 1B). As a result, the changing of miRNA-mediated regulation in two types of mRNAs resulted in different functional consequences (right panel of Figure 1), which could further affect phenotype and disease.

### 2.2. Experimental Evidence Supporting the Model

The experimental evidence to support the model is very limited. One is that a synonymous mutation (c.313C>T) in IRGM removed the miR-196 regulation, which resulted in decreased IRGM wild type variant (c.313C) but not the mutant allele (c.313T). As a result, the miRNA-mediated alteration in IRGM regulation affects the efficacy of autophagy and then Crohn’s disease risk [16].

Besides the experimental evidence described above, in the following we presented more evidence from large-scale analysis of genomic data.

### 2.3. Selection Signal Evidence from the Integrated Haplotype Score to Support the Model

**Figure 2:**
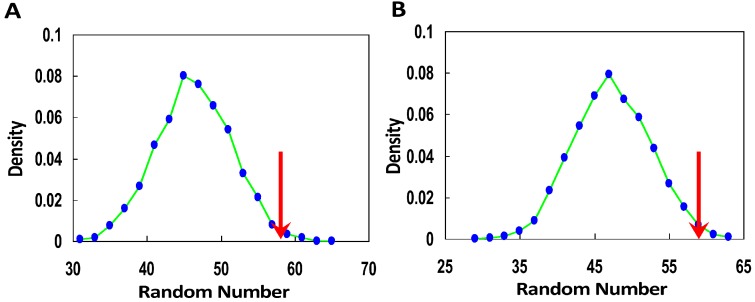
Evidence for the model from large-scale analysis of SNPs under positive selection (**A**) and SNPs associated with disease from GWAS (**B**). The green curves represent the distributions of random number of synonymous SNPs changing miRNA regulation under recent positive selection (**A**) and those associated with disease (**B**). The red arrows represent the real number of synonymous SNPs changing miRNA regulation in cases of A and B, respectively.

If the presented miRNA-mediated gene regulation model is true, that is, if the synonymous SNPs that change miRNA regulation could result in functional consequences and disease risk, these SNPs will tend to be under selection compared with those not changing miRNA regulation. To confirm this, we analyzed the integrated Haplotype Score (iHS), a statistic used to detect evidence of recent positive selection at a locus [18]. We obtained the iHS data for three populations, ASN (combined Japanese from Tokyo, Japan, and Han Chinese from Beijing, China), CEU (Utah residents with Northern and Western European ancestry from the CEPH collection), and YRI (Yorubans from Ibadan, Nigeria). Using TargetScan [19], a popular tool for miRNA binding site prediction, we identified the genome-wide aberrant miRNA binding sites (add/remove miRNA binding) within locus of synonymous SNPs. The result showed that 45.9% of the total synonymous SNPs could result in adding or removing at least one miRNA regulation. Furthermore, as expected, the synonymous SNPs changing miRNA regulation tend to have greater absolute iHS score than those not changing miRNA regulation (*p* = 0.03, 0.03 for ASI, YRI, respectively, Wilcoxon test). However, we did not observe a significant result in the CEU population. In addition, we obtained consistent results by analysis of Fisher’s tests (Table 1). For example, for the 21,296 synonymous SNPs changing miRNA regulation which include 3270 SNPs with iHS value, there are 58 SNPs under strong recent positive selection (absolute iHS ≥ 2.5) for the Asian population, which is significantly higher than the synonymous SNPs not changing miRNA regulation (odds ratio, OR = 2.0, *p* = 4.7 × 10^−4^, Fisher’s exact test, Table 1) and than the expected number in randomization test (expected number = 47, *p* = 0.02, randomization test; Figure 2A). In addition, we observed a significantly higher percentage of SNPs under recent positive selection for African population (*p* = 0.02, Table 1) but did not observe significant results for European population (*p =* 0.45). These results suggest that the synonymous SNPs changing miRNA regulation indeed tend to be more under recent positive selection than those not changing miRNA regulations.

**Table 1:** Statistics of synonymous SNPs in the context of recent positive selection, gene expression, and miRNA-mediated gene regulation.

Population	*p*-Value	Recent Selection	A	B
ASI	4.7 × 10^−4^	C	58	44
		D	3212	4972
YRI	0.02	C	79	63
		D	4449	5259
CEU	0.46	C	51	71
		D	3954	4765
Genotype & Expression	0.02	E	59	45
		F	21,237	25,698

A—changing miRNA-mediated gene regulation; B—not changing miRNA-mediated gene regulation; C—under recent positive selection; D—not under recent positive selection; E—association with disease; and F—no association with disease.

### 2.4. Gene Expression Evidence to Support the Model

**Figure 3:**
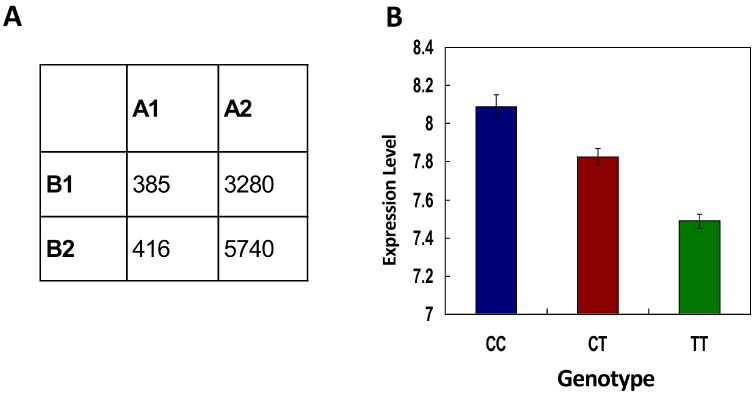
Evidence for the model from large-scale analysis of correlation between genotype and gene expression. The category table represents the relationship between synonymous SNPs changing miRNA regulation and gene expression (**A**). A1—Significant-correlation between gene expression and genotype (*p* ≤ 0.05); A2—Non-significant-correlation between gene expression and genotype (*p* > 0.05); B1—Genes that host synonymous SNPs changing miRNA regulation; and B2—Genes that do not host synonymous SNPs changing miRNA regulation; The sub-figure (**B**) shows the relationship between the genotype at the synonymous SNP (rs2247761) and gene TRIM4 expression level.

The truth that the synonymous SNPs changing miRNA regulation are more functional implies that these mutations prefer to result in functional consequences. A recent study presented the transcriptome of lymphoblastoid cell lines (LCLs) from 60 CEU individuals [20], which makes it possible to investigate the effect of synonymous SNPs on gene expression when combining these data with genetic variants from the HapMap project. To identify the genes that are affected by synonymous SNPs, we performed spearman rank correlation analyses of genotypes to gene expression data as described in the original article [20]. As a result, we found that the synonymous SNPs changing miRNA regulation significantly prefer to affect gene expression. For the genes that host synonymous SNPs changing miRNA regulation, 10.5% (385/3665) of them have a significant correlation between the genotype and the expression level (*p* ≤ 0.05); whereas the percentage decreases to 6.8% (416/6156) for the genes that do not host synonymous SNPs changing miRNA regulation (OR = 1.6, *p* = 6.0 × 10^−11^, Figure 3A), which could be resulting from a variety of factors such as changed *trans*-/*cis*- gene regulation and changed mRNA secondary structure. For example for the SNP rs2247761, the host gene (TRIM4) with allele “T” adds the regulation-mediated by miR-335 and miR-3123. There is a significant correlation between TRIM expression and the rs2247761 genotype (*p =* 1.9 × 10^−11^, Figure 3B). These results indicated that synonymous SNPs changing miRNA regulation could result in differential gene expression and therefore finally have functional consequences. In addition, for the 58 synonymous SNPs changing miRNA regulation and under positive selection in ASI, one (rs7644369, absolute iHS = 2.91) of them also has a significant correlation between the genotype and the expression level of its host gene (EPHB1). The change from the C allele to the T allele of this SNP deletes the regulation of EPHB1 by miR-4434 and miR-4516. The reason why only one SNP is overlapped between SNPs under recent positive selections and SNPs showing correlations with gene expression level could be that these two issues represent different functional aspects and they do not have significant cross talks in this case.

### 2.5. Genome-Wide Association Loci Evidence for Human Disease and Trait to Support the Model

As described above, synonymous SNPs changing miRNA regulation tend to be functional and affect expression of target genes, thus they could be implicated in disease risk and trait. To confirm this, here using SNPs for human disease and trait from the NHGRI GWAS Catalog [6] we investigated whether there is a preference of synonymous SNPs changing miRNA regulation on human disease and traits compared with those not changing miRNA regulation. As a result, for the 21,296 synonymous SNPs changing miRNA regulation, 59 SNPs have associated disease; whereas for the 25,743 synonymous SNPs not changing miRNA regulation, only 45 SNPs have associated disease. Indeed, the results showed that the synonymous SNPs changing miRNA regulation significantly prefer to be implicated in human diseases and traits (OR = 1.6, *p* = 0.02, Fisher’s exact test, Table 1). This significance was further confirmed by a randomization test (*p =* 0.02, Figure 2B). This finding indicates that synonymous SNPs could have functional consequences and determine disease risk and trait.

To further solidify this finding, we next repeated the above process using another disease-related genomic variation dataset, ClinVar. As a result, for the 21,296 synonymous SNPs changing miRNA regulation, 1720 SNPs are associated with disease; whereas for the 25,743 synonymous SNPs not changing miRNA regulation, 1663 SNPs are associated with disease, indicating that the synonymous SNPs changing miRNA regulation significantly prefer to implicate in human disease and trait (OR = 1.3, *p* = 9.0 × 10^−11^, Fisher’s exact test).

### 2.6. Extension of the Model

Besides synonymous SNPs, there are a lot of non-synonymous SNPs in coding regions, which attract the main focus from researchers during the past time because they can change amino acid composition and therefore were considered to have the potential to result in functional consequences. For example, many methods were developed to analyze whether a non-synonymous SNP is deleterious [21]. As a result, more efforts were devoted to the predicted deleterious mutations and the benign were often neglected. However, considering the framework we presented above, any mutations changing or not changing amino acid composition could lead to alteration of miRNA-mediated gene regulation, which may finally result in functional consequences. Interestingly, based on GWAS data [6] and the predicted results for non-synonymous SNPs by SIFT [21], we found that there are 226 disease related SNPs in the 33,294 predicted benign SNPs and there are 48 disease related SNPs in the 7442 predicted deleterious SNPs. We did not find differences for disease risk between the deleterious non-synonymous SNPs and the benign non-synonymous SNPs (OR = 0.95, *p* = 0.41, Fisher’s exact test), suggesting that the benign mutations could be equally important to the so-called deleterious. This finding further implies that the traditional researches neglect some important mechanism, for example miRNA-mediated regulation by binding coding regions of target genes. Thus here we extended our model to the non-synonymous SNPs. The results showed that 45.7% (22,289/48,768) of non-synonymous SNPs changed miRNA-mediated gene regulation, suggesting that the non-synonymous mutations could have general and important effects on protein function and disease risk through miRNA-mediated gene regulation. The non-synonymous SNPs changing miRNA regulation also showed distinct features compared with those that do not change miRNA regulation. The non-synonymous SNPs changing miRNA regulation also tend to have greater |iHS| value (*p* values for |iHS| in ASI, CEU are 0.008 and 0.01 respectively). The result for YRI is not significant (*p =* 0.5). These results suggest that they tend to be more under recent positive selection. There are 159 SNPs associated with disease in the 22,289 non-synonymous SNPs changing miRNA regulation; whereas there are only 118 SNPs associated with disease in the 26,479 non-synonymous SNPs not changing miRNA regulation, suggesting that the non-synonymous SNPs changing miRNA regulation prefer to implicate in human disease and trait (OR = 1.6, *p* = 5.9 × 10^−5^) compared with the non-synonymous SNPs that do not change miRNA regulation. These results suggest that miRNA-mediated regulation aberration could be a putative mechanism for the contribution of non-synonymous SNPs to functional consequences and disease risk. This further means that under the context of miRNA regulation, the so-called benign non-synonymous SNPs predicted by traditional analysis tools should be not neglected and should get equal consideration.

## 3. Discussion

In summary, we hypothesize that the synonymous SNPs could contribute to functional consequences and disease risk through aberration of miRNA-mediated gene regulation. This model can also be extended to other types of mutations in coding regions, for example the non-synonymous SNPs. This putative model could bring benefits in the following aspects. First, it provides a possible explanation for the contribution of synonymous SNPs to disease risk. Second, it could affect the opinion that many efforts should be focused on the non-synonymous SNPs especially the so-called predicted deleterious ones. The synonymous SNPs and the benign non-synonymous SNPs should be equally considered as well as the deleterious non-synonymous SNPs in the future. Third, it may have an effect on molecular evolution. For many concepts and analysis in evolution, the great majority, if not all, of the synonymous SNPs are frequently taken as neutral control [3]. This putative model, however, could present a possibility for the contributions of synonymous SNPs to functionality, suggesting that many synonymous SNPs may be not neutral. Therefore, the concepts and analysis that take synonymous SNPs as neutral control should be modified. Of course, future challenges exist. Understanding the exact molecular mechanism about how miRNAs regulate target genes by non-canonical binding sites will be critical for predicting the coding region SNPs that change miRNA regulation. In addition, tools for identifying miRNA regulation related deleterious SNPs in coding regions are also needed. Moreover, it is well known that different miRNA target prediction tools (e.g., TargetScan and miRanda) have very limited consistence in the predicted targets. The consistence of the predicted target sites is much lower. Therefore, it is not surprising that the consistence of the predicted synonymous SNPs changing miRNA regulation is very low. Further miRanda (another popular miRNA target prediction tool)-based analysis showed that the overlapped SNPs changing miRNA regulation by TargetScan and by miRanda is low. However, the results using miRanda-based miRNA targets remain mainly unchanged. For example, the *p*-values for ClinVar disease SNP, GWAS disease SNP, ASI, YRI are 5.356 × 10^−13^, 0.04, 0.06, and 0.06. Although the latter two *p*-values are a little bit greater than 0.05, they have a high tendency of significance. For the CEU, miRanda-based analysis is not significant (*p =* 0.76), which is also consistent with TargetScan-based analysis. These results further support this putative model but also imply that both miRNA target prediction tools covered but did not cover all true miRNA-changing SNPs. Therefore, it is believed that the improvement of miRNA target prediction tools will improve the prediction accuracy of synonymous SNPs changing miRNA regulation.

## 4. Experimental Section

### 4.1. Data Used in this Study

The common synonymous SNPs and the non-synonymous SNPs (dbSNP 137) are obtained from UCSC genome browser (http://genome.ucsc.edu/). Therefore, all of these SNPs are located only in CDS. We used the global human synonymous SNPs and non-synonymous SNPs in this study. As a result, we obtained 47,039 synonymous SNPs and 48,768 non-synonymous SNPs, respectively.

The iHS score from three populations, ASN (combined 45 Japanese from Tokyo, Japan, and 45 Han Chinese from Beijing, China), CEU (90 Utah residents with Northern and Western European ancestry from the CEPH collection), and YRI (90 Yorubans from Ibadan, Nigeria) is obtained from the haplotter website (http://haplotter.uchicago.edu/). The transcriptome of lymphoblastoid cell lines (LCLs) from 60 CEU individuals and their genotype data are obtained from the lab website of Emmanouil T. Dermitzakis (http://funpopgen.unige.ch/data/ceu60). The human disease and trait loci are obtained from the NHGRI GWAS Catalog (http://www.genome.gov/gwastudies/) and the ClinVar database (http://www.ncbi.nlm.nih.gov/clinvar/).

### 4.2. Tools for Data Analysis

SNPs which are associated with predicted miRNA binding sites include the SNP sites changing miRNA regulation and the SNP sites that do not change miRNA regulation but affect the miRNA-target binding affinity. Because it is difficult to evaluate the significance of the changed miRNA-target binding affinity by synonymous SNPs, we only focused on SNP sites changing miRNA regulation in this study. Here we predicted the synonymous mutation sites and the non-synonymous mutation sites that change miRNA-mediated gene regulation using TargetScan (http://www.targetscan.org/) and miRanda (http://www.microrna.org/microrna/getDownloads.do). For this purpose, we first generated two sequences based on the two alleles of a given SNP, respectively. Because TargetScan runs based on the seed region (2~8 nt) of miRNAs, the sequence we generated is 31 nt in length and the central nucleotide is the corresponding allele. For each miRNA, we next used TargetScan with default parameters to scan the generated sequences. After comparing the binding miRNAs for the sequences generated from one SNP, we outputted the predicting list of candidate synonymous SNPs and non-synonymous SNPs changing miRNA regulation. As a result, we obtained 21,237 synonymous SNPs and 22,289 non-synonymous SNPs changing miRNA regulation, respectively. Moreover, we presented the two datasets at http://www.cuilab.cn/smir. Statistical analysis such as Sperman’s correlation and Fisher’s exact test were performed using R, a free statistical tool (http://cran.r-project.org/). The permutation tests were performed using an in-house java program.

## 5. Conclusions

In conclusion, we performed a large-scale analysis for human synonymous SNPs changing miRNA-mediated regulation and synonymous SNPs not changing miRNA-mediated regulation. The result showed that the synonymous SNPs changing miRNA-mediated regulation are significantly more associated with functionality and disease susceptibility.
